# Mechanisms for Modulating Anoikis Resistance in Cancer and the Relevance of Metabolic Reprogramming

**DOI:** 10.3389/fonc.2021.626577

**Published:** 2021-03-29

**Authors:** Funmilayo O. Adeshakin, Adeleye O. Adeshakin, Lukman O. Afolabi, Dehong Yan, Guizhong Zhang, Xiaochun Wan

**Affiliations:** ^1^Guangdong Immune Cell Therapy Engineering and Technology Research Center, Center for Protein and Cell-Based Drugs, Institute of Biomedicine and Biotechnology, Shenzhen Institutes of Advanced Technology, Chinese Academy of Sciences, Shenzhen, China; ^2^University of Chinese Academy of Sciences, Beijing, China

**Keywords:** ECM detachment, anoikis, anoikis resistance, metabolism, tumor metastasis

## Abstract

The attachment of cells to the extracellular matrix (ECM) is the hallmark of structure–function stability and well-being. ECM detachment in localized tumors precedes abnormal dissemination of tumor cells culminating in metastasis. Programmed cell death (PCD) is activated during tumorigenesis to clear off ECM-detached cells through “anoikis.” However, cancer cells develop several mechanisms for abrogating anoikis, thus promoting their invasiveness and metastasis. Specific factors, such as growth proteins, pH, transcriptional signaling pathways, and oxidative stress, have been reported as drivers of anoikis resistance, thus enhancing cancer proliferation and metastasis. Recent studies highlighted the key contributions of metabolic pathways, enabling the cells to bypass anoikis. Therefore, understanding the mechanisms driving anoikis resistance could help to counteract tumor progression and prevent metastasis. This review elucidates the dynamics employed by cancer cells to impede anoikis, thus promoting proliferation, invasion, and metastasis. In addition, the authors have discussed other metabolic intermediates (especially amino acids and nucleotides) that are less explored, which could be crucial for anoikis resistance and metastasis.

## Introduction

Cell–cell adherence and their interaction with the extracellular matrix (ECM) are imperative for the growth and survival of cells. The ECM is a three-dimensional scaffold that provides the required critical biochemical and mechanical signals for tissue formation, growth, differentiation, and homeostasis (1). It is composed of varying biomolecules, such as glycosaminoglycans, proteoglycans, and fibrous proteins (1, 2). Adhesion receptors, such as integrin, cadherin, selectin, and immunoglobulin-like cell adhesion molecules, mediate the attachment of cells to the ECM (3). The meshwork between the biomolecules and cell adhesion molecules in the ECM regulates homeostasis. Aberrations in the ECM often lead to severe dysfunction and even death, both at the microscopic and organism levels, which have been implicated in diverse mammalian diseases, including cancer (4, 5).

Cell death is a vital occurrence as it facilitates the development and differentiation of tissues, clearance of harmful or damaged cells, and maintenance of homeostasis (6, 7). Some studies had highlighted that cell death a regulated event and categorized it as programmed cell death (PCD) and non-PCD based on the biochemical and molecular signaling activities within and without cell milieu (8–10). PCD includes both apoptotic and non-apoptotic cell death. Apoptotic cell death (apoptosis and anoikis) mainly occurs through the activation of caspase while non-apoptotic cell deaths are independent of caspase (9, 10). Furthermore, non-apoptotic cell death includes entosis, methuosis, paraptosis, mitotopsis, parthanatos, ferroptosis, pyroptosis, netosis, necrosis, and autophagic cell death as mentioned in the previous studies (9, 10). On the other hand, non-PCD, also known as accidental cell death, is a type of death resulting from an unpredicted or unforeseen cell injury (8, 9).

The disruption in cell–cell attachment or cell-ECM attachment leads to a form of apoptotic cell death, which is termed as “anoikis” (11). Anoikis is a mechanism for the elimination of misplaced or detached cells under physiological or pathological conditions, which facilitate tissue homeostasis (12). Anoikis in cancer cells retards metastasis of cells to other sites; however, this is often not the case due to cancer cells becoming anoikis resistant. In addition to cancer, anoikis occurs in other pathological conditions such as diabetes and cardiovascular disorders (13). It ensures clearance of detached cells and this could be detrimental if excessively activated in diabetes and cardiovascular disease (14, 15). The initiation of anoikis is facilitated through the intrinsic (mitochondrial approach) and extrinsic (death receptor approach) caspase activation similar to the apoptotic cascade of endonucleases activation, DNA damage, and cell death (11, 16). Anoikis resistance occurs occasionally when detached cells circumvent death signaling pathway, enabling survival of cells as a result of several biochemical and molecular alterations within the cell milieu. These changes are the hallmark of invasiveness, metastasis, therapy resistance, and relapse of cancer cells.

Cancer is the second leading cause of death (17), most of which is not due to localized tumors but a result of metastasizing cells within the tumor mass. Cancer cells metastasize after successive processes of detachment from one another or the ECM, migrating to distal points, promoting reattachment, and proliferation in the new site (18). Cancer cells employ several mechanisms for abrogating anoikis, thus promoting their invasiveness and metastasis. Cellular acidosis and the changes in reactive oxygen species (ROS) generation in cancer cells had a tremendous impact in promoting anoikis resistance (19–21). These changes promote oncogenic signals that induce pro-survival pathways, leading to stemness, proliferation, and invasion (19–21). Similarly, the expression of long non-coding RNA (lncRNA) correlated with anoikis resistance and metastasis in cancer (22, 23). Recent studies highlighted the role of metabolic alteration and signaling pathways driving metabolic processes as an alternative approach to target anoikis resistance in cancer cells (24–26). While nutrient uptake by cells is necessary to supply the energy required for cellular growth, abnormal metabolism in patients with cancer could lead to anoikis resistance and promote therapy resistance. Therefore, a holistic understanding of several mechanisms underlying anoikis resistance could help in delaying tumor progression and prevent metastasis.

This review aims to explore the current knowledge on apoptosis, the regulators of anoikis resistance [such as pH, oxidative stress, growth factors (GFs), cancer stem cells (CSCs), and lncRNA], the role of signaling pathways as “drivers” of anoikis resistance, and the relevance of targeting metabolism to ameliorate tumor metastasis. The authors also shared their perspective on potential targets for ameliorating metastasis and their insights on current therapies that sensitize cancer cells to anoikis.

## Anoikis: A Particular Type of Apoptosis

Biochemical and environmental changes within and outside the cell can activate cell death signals. Apoptosis is a form of regulated cell death involved in terminating the cell function to ensure homeostasis, normal growth, and the elimination of potential hazards (6, 7). Cell shrinkage, cell blebbing (plasma membrane protrusion), karyorrhexis (nucleus fragmentation), pyknosis (irreversible condensation of chromatin), and DNA fragmentation are some features of cells undergoing apoptosis (27–29). Prior to the aforementioned features, apoptosis can be initiated through intrinsic, extrinsic, or perforin/granzyme A/B pathways (16). All apoptosis initiation pathways are largely caspase dependent except granzyme A, which induces damage to the mitochondria as a committed step for apoptosis following cellular stress (30). Perforin/granzyme is a cytotoxic lymphocyte-mediated process. Granzyme B impaired tumor spread, migration, and invasion in human cancer cell lines while it induced anoikis in endothelial cells (ECs) through the modulation of cell adhesion (31). The authors identified that granzme B cleaved some ECM proteins, including laminin, fibronectin, and vitronectin, to induce detachment, anoikis, and inhibition of the migration (31).

The intrinsic pathway of apoptosis is a cellular stress-induced mitochondrial event. These stress signals could lead to permeabilization of the mitochondria or its swelling through the formation of membrane pores and the release of apoptotic proteins, such as cytochrome c, second mitochondria-derived activator of caspases (Smac/DIABLO), and serine protease (16). These proteins activate caspases for apoptosis in the mitochondria. Cytochrome c binds to an apoptotic protease activating factor-1 (Apaf-1) to form an apoptosome complex, which induces death signal spiraling pro-caspase-9 to caspase-9 and activates the effector caspase-3 and effector caspase-7. Smac/DIABLO and serine proteases promote apoptosis by antagonizing the inhibitors of apoptosis to further enhance the activity of effector caspase-3, effector caspase-7, and effector caspase-9 for killing the cells (32).

The extrinsic approach is mediated by tumor necrosis factors (TNFs) and the first apoptosis signal (Fas)-ligand (16). TNF binds to a TNF receptor (TNFR1) and propels the activation of caspase-8 for the cleavage of other effector caspases to activate apoptosis through TNF receptor-associated death domain (TRADD) and Fas-associated death domain (FADD) (16). Fas-ligand binds to an Apo-1 or CD 95-a transmembrane protein of the TNF family to form a death-inducing signaling complex (DISC). DISC activates caspase-8, which in turn activates effector caspase-3 and effector caspase-7 for cell death (33).

Anoikis is essentially an apoptotic event, which is activated to clear detached cells (34), and is a form of regulated cell death that utilizes both the intrinsic and extrinsic pathways. Similar to apoptosis, pro-apoptotic events are activated in the mitochondria or DISC formation to propel the activity of effector caspases for elimination of detached cells and restoration of tissue homeostasis.

## Regulators of Anoikis Resistance

**Figure 1:**
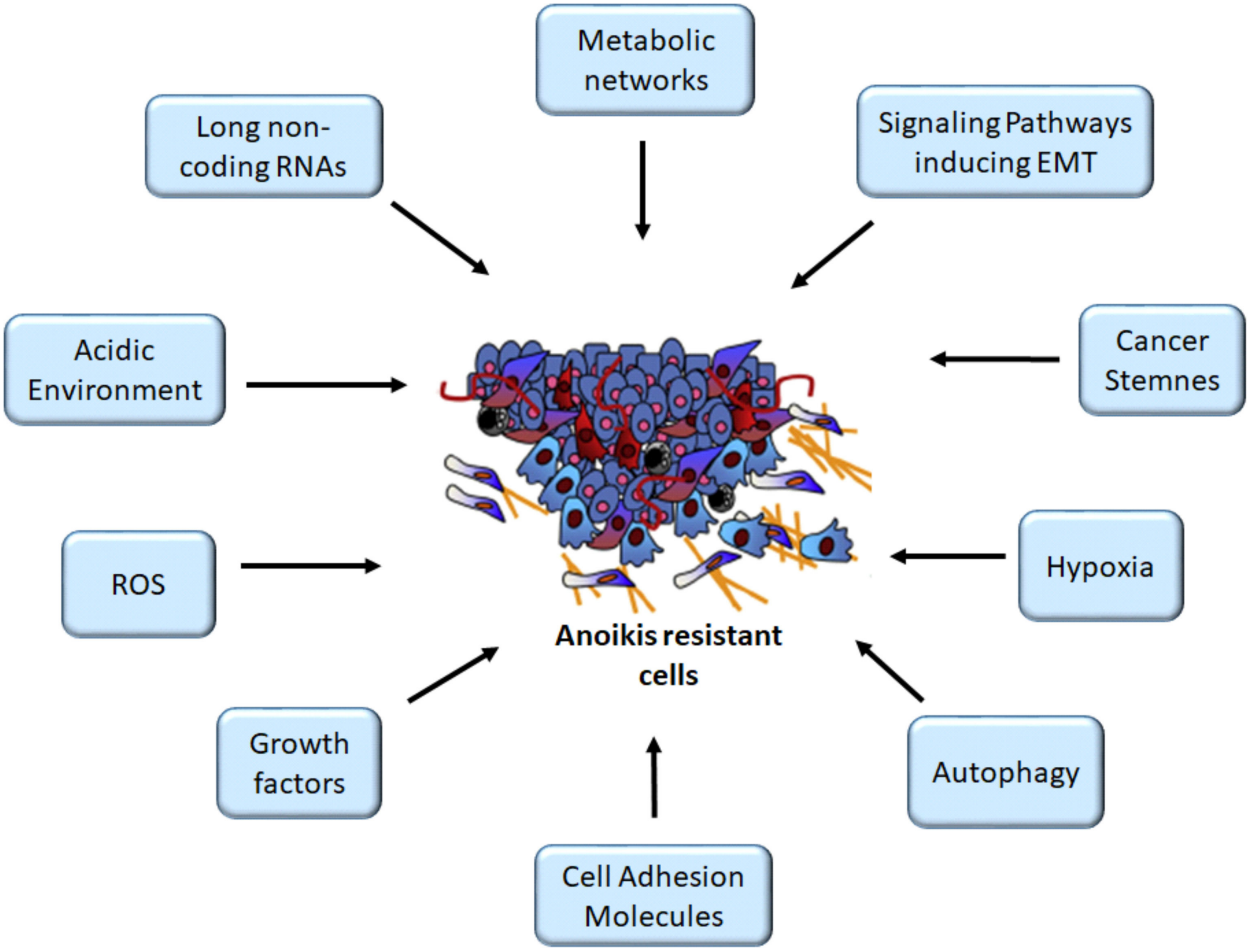
Mediators of anoikis-resistant cells. The figure depicts several factors altering anoikis mechanism, thus enhancing cancer cell survival following detachment, an important criterion for the initiation of metastasis cascade. ROS, reactive oxygen species. Image for anoikis resistant cells was adapted from (11).

**Figure 2:**
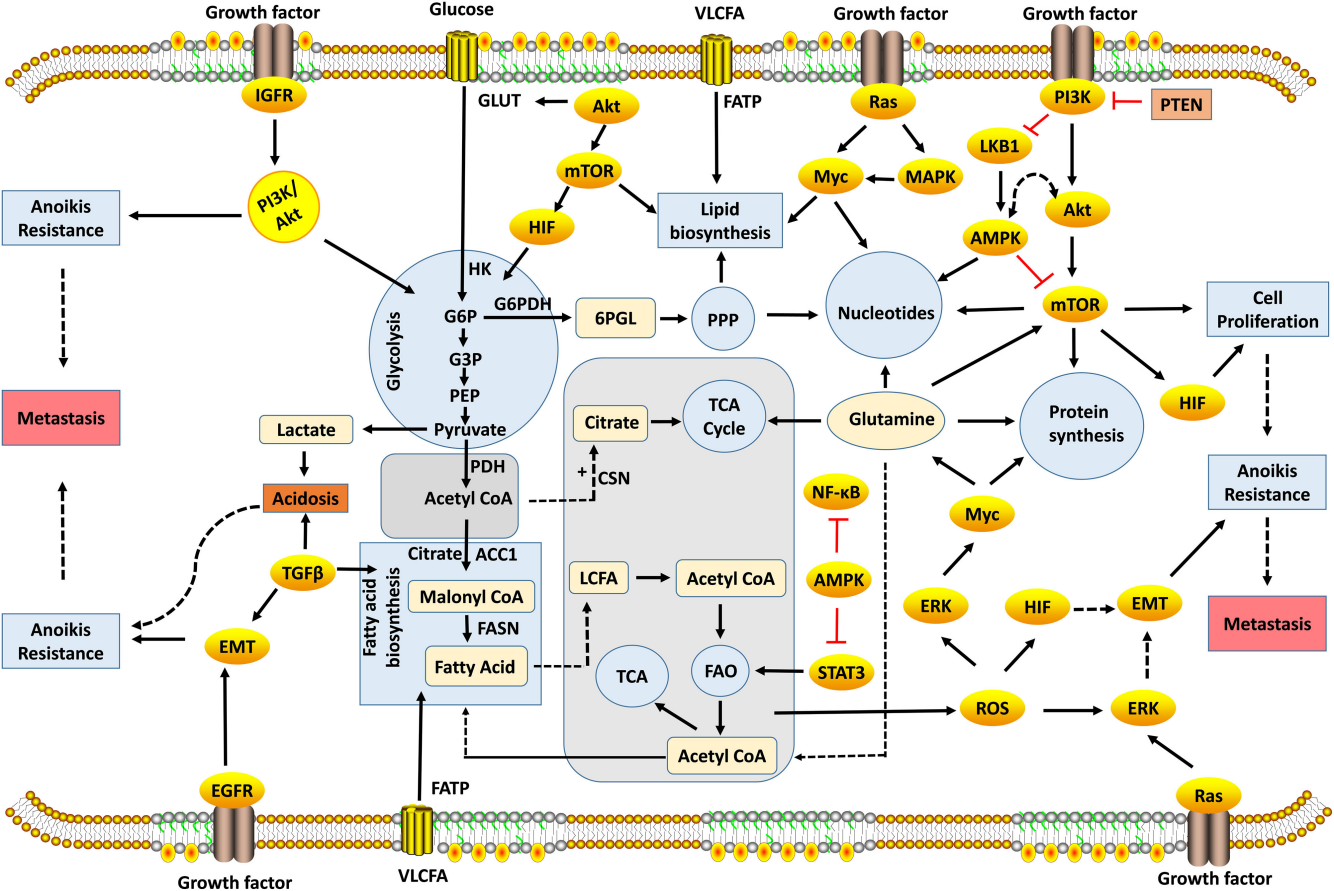
Metabolic and signaling networks involved in anoikis-resistance mechanism. Several metabolic intermediates generate the energy required for cellular growth and survival; oncogenic activation of signaling pathways reprogram cellular metabolism to promote anoikis resistance. Metabolic pathways occur in different compartments, which involve the biosynthesis or degradation of biomolecules to supply energy needs of the cell. Glycolysis, PPP, protein, fatty acid, and nucleotide biosynthesis take place in the cytosol, while TCA and FAO occur in the mitochondria. The decarboxylation of pyruvate to acetyl-CoA takes place in the mitochondrial matrix. These metabolic pathways are interrelated with acetyl-CoA, which acts a key substrate for connecting these pathways. Metabolism is regulated by rate-limiting enzymes that allow the cell to maintain cellular homeostasis. However, the dysregulation of genes encoding these enzymes leads to metabolic perturbation that drives anoikis resistance preceding tumor metastasis. 6PGL, 6-Phosphogluconolactone; AMPK, Adenosine Monophosphate Kinase; ACC1, Acetyl Carboxylase 1; ATP, Adenosine Triphosphate; CSN, Citrate Synthase; EGFR, Epidermal Growth Factor Receptors; EMT, Epithelial-Mesenchymal Transition; ERK, Extracellular-Signal-Regulated Kinase; FAO, Fatty Acid Oxidation; FASN, Fatty Acid Synthase; FATP, Fatty Acid Transport Proteins; G3P, Glyceraldehyde-3-Phosphate; G6P, Glucose-6-Phosphate; G6PDH, Glucose-6-Phosphate Dehydrogenase; GLUT, Glucose Transporters; HIF, Hypoxia-Inducible Factors; HK, Hexokinase; IGFR, Insulin-Like Growth Factor-1 Receptor; LKB1, Liver Kinase B1; MAPK, Mitogen-Activated Protein Kinase; mTOR, Mammalian Target of Rapamycin; NF-κB, Nuclear Factor-Kappa B; PDH, Pyruvate Dehydrogenase; PEP, Phosphoenolpyruvate; PI3K, Phosphatidylinositol 3-Kinase; PPP, Pentose Phosphate Pathway; PTEN, Phosphatase and Tensin; ROS, Reactive Oxygen Species; STAT3, Signal Transducer and Activator of Transcription 3; TCA, Tricarboxylic Acid; TGF-β, Transforming Growth Factor-Beta; VLCFA, Very Long-Chain Fatty Acid.

The evasion of anoikis in detached cells from localized tumors results in uncontrolled growth of these cancer cells in other sites. Studies have identified several factors, such as cell adhesion molecules, growth proteins, oxidative stress, stemness, autophagy, non-coding RNAs, and signaling pathways, for modulating anoikis resistance (Figures 1, 2). In the present study, we discuss some of these factors and their roles in mediating anoikis resistance, which are summarized in Table 1.

**Table 1:** Summary of the anoikis-resistance mechanism in cancer cells.

**Gene or pathways**	**Cancer type**	**Mechanism of action**	**References**
**Cell adhesion molecules**			
E-cadherin↓ N-cadherin↑	Laryngeal small cell carcinoma	Promotes anoikis resistance	(35)
Integrin αvβ3	Pancreatic	Activation of cSrc in a FAK-independent manner	(36)
Integrin β1↑	HNSCC	Modulates the MMP-2 activation to induce metastatic potential	(37)
Integrin α2β1↑	Prostrate	Activation of p38-MAPK-MMP-1	(38)
E-selectin↑		Promotes shear-resistant adhesion to endothelial cells	(39)
	Lymphoma	Activation of E-selectin-FAK signaling	(40)
Syndecan-2↑	Melanoma	Activates PI3K/Akt and ERK signaling	(41)
**pH**			
Extracellular pH↓	Melanoma	Enhanced motility *via* EGFR-Akt pathway	(42)
	Human pharyngeal squamous cell carcinoma, cervical and colorectal	Activation of TGF-β2 and lipid droplet formation for β-oxidation	(43)
**ROS**			
NOX4↑	Gastric and lung	Induction of EGFR signaling	(44, 45)
Mitochondrial ROS↑	Cancer stem cells	Activates MAPK and regulates EMT for cancer invasion and metastatic potential	(19)
ROS↓	Glioblastoma	Upregulation of HIF	(46)
MnSOD	Breast and nasopharyngeal	Promotes glucose oxidation and β-catenin signal	(47, 48)
**Growth factors**			
SRC-3Δ4/EGFR↑	Breast	Interaction with EGFR and FAK signaling	(49)
IGF/IGF1R	Breast	Activation of PI3K/Akt pathway	(50)
VEGF-A/VEGFR2↑	Ovarian	Initiation of autocrine VEGF-A/KDR loop	(51)
FGF-19 FGFR4	Breast	Activation of PI3K/Akt pathway	(52)
PDGF-BB	Pancreatic	EMT upregulation	(53)
BDNF/TrkB↑	Cervical	Activation of PI3k/Akt pathway	(54)
TrkB	Endometrial carcinoma	EMT activation *via* reduced E-cadherin and increased N-cadherin	(55)
	Renal cell carcinoma	Activation of PI3k/Akt pathway and MEK/ERK	(56)
EGFR-integrin axis		Blockade in Bim expression	(57)
HCRP-1↓	Colon	Upregulates EGFR activity to inhibit Bim expression	(58)
PRP4K↓	Ovarian	EGFR upregulation and endosomal signaling	(59)
EGF	HNSCC	Induced the expression of ANGPTL4 Regulates MMP1 *via* integrin β1/c-Jun signaling	(60)
**Long non-coding RNA (lncRNA)**			
ANRIL↑	Glioma	Downregulate p21 and caspase-3/8/9 activity Increase the expression of Bcl-2, CDK2, c-Myc, and p-Akt Activates Twist1 and c-jun transcriptional activity	(22)
MALAT1↑	Ovarian	Mediate the expression of RBFOX2 and KIF1B	(61)
LINC00958↑ LINC01296↑	Bladder	Induce oncogenic drivers and initiation of metadherin	(23)
**Cancer stemness**			
CD44^+^/CD24^−^↑ ALDH1↑	Breast	Induced phosphorylation of STAT3	(62)
MDA-9-GSC↑	Glioma	Inhibits autophagy Repress EGFR and Bcl-2 signaling	(63)
CMA↓	Embryonic stem cells	Increase activity of IDH1/2 necessary for the production of α-ketoglutarate Promotes renewal	(64)
**METABOLIC-RELATED SIGNALING PATHWAYS**			
**PI3K/Akt**			
PI3K/Akt↑	Breast	Inactivation of PTEN	(65)
	Osteocarcinoma	High expression of ID1 and activation of Src	(66, 67)
	HCC	Caveolin 1 induced the overexpression of IGF-1R	(68)
	Prostate	Downregulation of miR-133a-3p	(69)
Akt↑	Osteosarcoma	Induced stem cell-derived IL-8	(70)
RAS/ERK/PI3K/Akt↑	Hepatoma	Promoted anoikis resistance and cell invasiveness	(71)
P13K↑	Melanoma	Upregulation of Syndecan-2	(41)
**AMPK**			
AMPK↑	Lung	Activation of PLAG1-GDH1/CAMKK2 axis Repression of mTOR activity	(72)
	HCC	Trim31 suppresses p53 activation	(73)
	Breast	Ser116 phosphorylation of PEA15	(74)
		Suppresses mTORC1 pathway	(75)
		Repression of Akt	(76)
AMPK↓	cholangiocarcinoma	Inhibit NF-κB and STAT3	(77)
**HIF**			
HIF1α↑	Glioblastoma	Low ROS	(46)
HIF-1↑	Gastric	Upregulated ANGPTL4	(78)
		Inhibition of integrinα5	(79)
	Breast	Inactivation of Bim Phosphorylation of ERK and Akt	(80)
**STAT**			
STAT4↑	Ovarian	Wnt7a signaling triggered CAFs	(81)
STAT3↑	Pancreatic	Bcl-2 activation	(82)
	Cervical	Loss of Erbin	(83)
	Glioma	Induce MMP-2 expression thereby activate 1L-6 and α5β1	(84)
	TNBC	Promote stemness, MMP9, and MMP-2 expression	(62)
**Rho GTPase**			
Rac1 ↑	Breast cancer	Enhanced transcription of estrogen receptor-alpha	(85)
RhoA, Rac1, and Cdc42↓	Prostate Cervical Breast Colorectal	Inhibited cell migration Altered cell morphology *via* actin polymerization	(86)
Rho-associated kinases ↓	medulloblastoma	Impaired TNFα *via* NFkβ, TGF-β, and EMT Downregulated key proteins in the Rho-associated kinase pathway	(87)
**METABOLIC PATHWAYS**			
**Glucose metabolism**			
Glycolysis↑	TNBC	Decreased OCR Increased ECAR Induce CSC population	(88)
Glucose↓	Breast Pancreatic	Activates PKA Enhanced autophagy Activation of Src Promotes glutamine metabolism ER stress attenuation	(89)
Glycolysis↑	Breast	Promotes PPP Enhanced FAO Increased ROS and ATP generation	(90)
Glucose uptake↑	Breast Colon	Upregulation of GLUT1 Enhanced PPP Increase ATP production Induced SGK-1 expression	(91)
**Protein metabolism**			
CCDC178↑	HCC	Activates ERK/MAPK signaling pathway	(24)
PPAT↑	SCLC	Elevates c-Myc	(92)
GDH1↑	Glioblastoma	EGFR phosphorylation of ELK1 Activation of the MEK/ERK pathway	(25)
Asparagine bioavailability↑	Breast	Upregulates ASNS Regulates proteins that promote EMT	(93)
**Fatty acid metabolism**			
FASN↑	Osteosarcoma	Activation of p-ERK1/2 and Bcl-xL	(94)
CPT1↑	Colon	Activate FAO	(95)
AKR1B10↑	Breast	Activate FAO	(96)
CDCP1↑	TNBC	Drives FAO and OXPHOS Reduce ACSL activity in the cytoplasm Increased FA utilization in mitochondria	(97)
FA uptake↑	Human pharyngeal Squamous cell carcinoma, cervical and colorectal	Activation of TGF-β2 and FAO	(43)
**Nucleotide metabolism**			
Rgnef↑	Ovarian Colon	Interaction with FAK Activation of NF-κB and RhoA pathways Reduced ROS production	(98–101)

## Cell Adhesion Molecules

The role of adhesion molecules in ECM stabilization is critical for anoikis, and changes in these molecules could confer anoikis resistance, which is a prerequisite for metastasis. A recent study on laryngeal squamous cell carcinoma reported that E-cadherin and N-cadherin, which are markers of an epithelial-mesenchymal transition (EMT—a transformation predisposing cells to adopt an anoikis-resistance phenotype) were downregulated and upregulated, respectively, thus contributing to tumor progression (35). Integrin αvβ3 is involved in the transduction of antiapoptotic signals by facilitating anchorage-independent growth (AIG) and metastatic potential of pancreatic cancer through the activation of cSrc in a focal adhesion kinase (FAK)—independent manner (36). Head and neck squamous cell carcinoma (HNSCC)—expressing high-integrin β1 was correlated with metastatic potential, a low survival in patients by modulating metalloproteinase-2 (MMP-2) activation (37). Similarly, a study reported that a docetaxel resistant, high-integrin α2β1-expressing prostate cancer cell possessed an increased invasive migratory capability but a retarded proliferation. However, low α2β1-expressing prostate cancer cells had higher proliferation and compact spheroids with less migration potential (38). These authors demonstrated that high-integrin α2β1 invasive capability was a result of activation of the p38-MAPK-MMP-1 signaling pathways. Similarly, the treatment of a breast cancer cell line (MCF-7) with lectin, a glycoprotein, induced anoikis through the reduction of integrin and the impairment of integrin-FAK signaling (102).

Kang et al. reported that soluble E-selectin promoted anoikis resistance and migration through its interaction with CD44 in breast cancer cell lines [melanoma differentiation-associated (MDA)-MB-231 and MDA-MB-468 cell lines] to promote shear-resistant adhesion to EC and the circulation of tumor cells, thus promoting metastasis (39). High E-selectin with no significant changes in intercellular adhesion molecule-1 (ICAM-1) and vascular cell adhesion molecule-1 (VCAM-1) were observed in the circulation of EC-expressing B-cell lymphoma-2 (Bcl-2) (40). Employing neutralizing antibodies against E-selectin, ICAM-1 and VCAM-1 revealed that anti-E-selectin was able to block tumor cell binding, while anti-ICAM-1 and anti-VCAM-1 failed to significantly block tumor cell binding (40). This study showed that the coculturing of tumor cells with Bcl-2-expressing EC conferred anoikis resistance on the tumor cells *via* the activation of E-selectin-FAK signaling. However, blocking of the E-selectin or FAK restored anoikis sensitivity in cancer cells cocultured with EC (40). On the other hand, Roland et al. studied a positive correlation of ICAM-1 expression with cancer cell malignancy (103). Cell surface proteoglycans (such as the Syndecan family) participated in cell adhesion (104), anoikis-resistant EC was showed to upregulate Syndecan-4 in comparison to the parent EC through an increased heparanase expression (105). Perlecan, a heparan sulfate proteoglycan in the ECM, is involved in cascades of the metastasis process (106). Treatment of anoikis-resistant EC with trastuzumab (an anticancer drug) significantly reduced Syndecan-4 and perlecan expression, which impaired cell proliferation and invasion (107). The roles of some adhesion molecules in mediating anoikis resistance and metastasis were previously reviewed by other authors (106, 108, 109). However, further elucidation of the interactions of different adhesion molecules is inherent in understanding the dynamics of ECM reconstruction in modulating anoikis signals to better curb tumor metastasis.

## pH

The body homeostasis is dependent on the maintenance of physiological pH for different cells, tissues, and organs. Changes in pH were implicated in diseases (110). A recent study on melanoma identified that low extracellular pH promoted anoikis resistance through an increased N-cadherin expression with an enhanced migrative ability through the activation of epidermal growth factor receptor (EGFR) and Akt pathways (42). Nevertheless, the involvement of acidic-adapted melanoma cells in the metastatic cascade is yet to be elucidated (42). Another study reported that the culturing of cervical, pharynx, and colorectal cancer cell lines in an acidic environment showed an increase in lipid droplet formation, which facilitated anoikis resistance and the survival of cancer cells (43). Conversely, the significance of buffering acidity in solid tumor-bearing mice *via* oral administration of bicarbonate in combination with an immune checkpoint blockade or adoptive cell therapy in different cancer models was reported to improve the treatment outcome and survival of the mice model (111). Taken together, these studies suggest that the acidic environment stimulates anoikis resistance and tumor progression (Figure 1). Proper management of pH in solid tumors could be a promising approach to circumvent anoikis resistance. Therefore, future research can focus on the manipulation of pH to regulate the transformations conferring anoikis resistance in tumor cells.

## Reactive Oxygen Species

The production of ROS by tumor cells is indicative of oxidative stress. There are contrasting views on the role of ROS during tumor growth, and it is yet to be fully understood how alteration in oxidative stress could contribute to anoikis. Two independent studies reported the generation of ROS as a result of the upregulation of NADPH oxidase 4 (NOX4) in gastric and lung cancer cells grown in suspension; this was observed to induce anoikis resistance and metastasis *via* EGFR signaling (44, 45). A recent study revealed an elevated ROS in CSCs as a metastatic trait to enhance EMT and the invasiveness of various previously studied cancer cell lines (19). On the contrary, low ROS content was observed in glioblastoma cultured in suspension with a concomitant increase in the expression of hypoxia-inducible factor (HIF) and was observed to be anoikis resistant (46). Likewise, two independent groups reported that the upregulation of manganese superoxide dismutase (an antioxidant that mops up ROS) promoted anoikis resistance and tumor metastasis in mammary epithelial cells and nasopharyngeal carcinoma, which suggested a high-level ROS capable of sensitizing the cells to anoikis upon their detachment (47, 48). The role of reactive radicals in cancer cannot be overemphasized and still requires further investigation in cancer from different origins to determine the specific effects of varying (low, moderate, or high) levels of ROS generated inside and outside the tumor environment. This may help to elucidate the overall ROS involvement in regulating anoikis resistance, thus providing novel means of targeting these species for better cancer therapy.

## Growth Factors

Growth factors are regulatory molecules, mostly proteins or steroids, capable of initiating cell growth, proliferation, differentiation, inflammation, immunity, tissue healing and survival. The activity of GFs is accomplished by the interaction of GFs with target receptors, which could facilitate or incapacitate their functions. GF receptors are transmembrane proteins that bind to a specific GF and convey signals to the intracellular space. The receptors include EGFR, insulin-like growth factor (IGF) receptor, vascular endothelial growth factor (VEGF) receptor, fibroblast GF (FGF) receptor, platelet-derived GF receptor, and neurotropin receptor (112). For instance, epidermal growth factor (EGF) is a ligand of EGFR, a proto-oncogene that activates downstream kinases, such as FAK, to regulate cell adhesion site dynamics, thus promoting cancer growth, migration, and metastasis (49). IGF receptors include IGF-1 receptor (IGF1R), IGF-2 receptor (IGF2R), and insulin receptor (INSR). IGF/IGF1R mediated anoikis resistance in estrogen-responsive breast cancer *via* the signaling of a phosphoinositide-3-kinase (PI3k)/Akt pathway (Figure 2) (50). The expression of IGF receptors was implicated in the progression, metastasis, and therapy resistance of cancer (113, 114). The expression of VEGF-A and its receptor (VEGFR2/KDR) in epithelial ovarian cancer facilitated anoikis resistance, and the targeting of VEGF-A using a neutralizing antibody or KDR using a specific inhibitor sensitized the cells to anoikis (51). The signaling of FGF receptor-19 (FGFR19) and FGFR4 enabled the survival of breast cancer cells lines co-expressing them *via* the activation of PI3K/Akt pathway, targeting FGFR4/FGF19-sensitized cancer cells to death (52).

Brain-derived neurotrophic factor (BDNF) is a member of the neurotropin family, which is critical for neuron development and regeneration (115). The BDNF interaction with its receptor tyrosine kinase B (TrkB) was associated with anoikis resistance in cervical cancer (54). BDNF/TrkB expression was high in the anoikis-resistant model and promoted proliferation *via* the phosphorylation of P13k/Akt pathway (54). The upregulation of TrkB was also reported to promote EMT and anoikis resistance in endometrial carcinoma (55). Also, the silencing of TrkB in anoikis-resistant renal cell carcinoma abrogated proliferation, invasion, and sensitized cells to chemotherapy (sorafenib) through inactivation of the PI3K/Akt pathway and MEK/ERK signaling pathway (56). In addition to specific receptors, GFs also interact with the ECM to implement their functions (116). FGF is secreted into extracellular space where the cells bind to heparin-like glycosaminoglycan in the ECM to affect the localization of FGF and signal transduction in target cells (117). EGFR–integrin interaction was shown to inhibit anoikis by regulating Bim, a pro-apoptotic protein during cell detachment from the ECM (57). Also, the loss of hepatocellular carcinoma receptor protein 1 (HCRP-1) upregulated EGFR activity in modulating the expression of Bim, thus facilitating anoikis resistance and metastasis of colon cancer (58). In the same vein, a reduced pre-mRNA splicing factor 4 kinase (PRP4K) upregulated EGFR, thus facilitating anoikis resistance and metastasis (59). A different study reported that EGF induced the expression of angiopoietin-like-4 (ANGPTL4) protein in HNSCC, thereby favoring anoikis resistance (60). EGF regulated the expression of MMP-1 *via* integrin β1/c-Jun signaling, thus enabling the invasiveness and metastasis of HNSCC (60). As previously discussed, the NOX4-induced ROS generation in the cancer-activated EGFR signaling fostered anoikis resistance and metastasis (44, 45). Under an acidic environment, the upregulation of transforming growth factor-beta 2 (TGF-β2) induced anoikis resistance by facilitating EMT and lipid metabolism (Figure 2 and Table 1) (43). Also, TGF-β stimulated FGFR isoform switch to facilitate EMT and cancer progression (118). The exposure of pancreatic adenocarcinoma to platelet-derived GF-BB was recently reported to promote anoikis resistance and cell migration through EMT (53). The interaction of GFs with pro-survival signaling pathways mediated anoikis resistance to promote oncogenic transformation and metastasis in bone sarcoma (119). The activity of GFs is important for changes in the ECM skeleton, which is capable of culminating in anoikis attenuation, uncontrolled growth, metastasis, and relapse in some cases. Melosky discussed the treatment strategies for EGFR mutations in non-small cell lung cancer (NSCLC) and highlighted the existing EGFR mutations with an emphasis to first, second, and third lines of treatment plans in NSCLC (120). Although this study only focused on EGFR mutations and therapies in NSCLC, future studies can explore a similar approach for EGFR mutant cancers or other GFs driving oncogenic transformations to improve the current therapies (Figure 2). Other authors had reviewed the role of GFs and their receptors in mediating cancer (121–123). Therefore, the evaluation and targeting of aberrantly expressed growth proteins interacting with oncogenic markers may improve understanding of the impact of GFs on anoikis resistance and cancer metastasis.

## Long Non-coding RNA

Long non-coding RNA (lncRNA) is composed of more than 200 nucleotides with no capability of being translated into proteins and is classified based on their location, mechanism of action, and the effects on DNA sequences. LncRNAs play diverse roles in epigenetic modification, protein synthesis, RNA transport, molecular signal regulation, among others, and have been implicated to be involved in anoikis resistance of cancer cells stemming toward metastatic phenotypes (124–126).

The antisense non-coding RNA in the INK4 locus (ANRIL) expression was found to be positively correlated with glioma grade in the tissues examined by Dai et al., and silencing of ANRIL in U251 cell lines induced anoikis and impaired cell proliferation (22). Similarly, metastasis-associated lung adenocarcinoma transcript 1 (MALAT1) was identified to be highly expressed in ovarian cancer cells, thereby promoting EMT, anchorage independence growth, and metastasis (61). The blockade of MALAT1 sensitized the cells to anoikis and inhibited the invasive potential (61).

Several lncRNAs were identified to be abnormally expressed in bladder cancer (23). However, LINC00958 and LINC01296 were upregulated, and their expression was observed to be correlated with anoikis-resistance fostering oncogenic signals, which were incumbent to the progression of bladder cancer (23). Although there are a myriad of lncRNAs (127), of which a few are associated with anoikis, it is, therefore, pertinent to explore how other aberrantly expressed lncRNAs which are yet to be identified in various cancers could contribute to anoikis or its resistance, thus furthering strategies to target cancer metastasis.

## Cancer Stemness and Autophagy

Cancer stem cells exhibit characteristics of both stem cell and cancer cell. In addition to self-renewal and differentiation capacities of stem cells, CSCs can become tumors when transplanted into an animal model. The stem cells from MDA-MB-453 and MDA-MB-231 were reported to be anoikis resistant while non-stem bearing cells were sensitive to anoikis (128). Coculturing of stem-like cells with non-stem-like cells conferred anoikis resistance on non-stem-like cells (128). A different study reported that an amplified expression of CD44^+^/CD24^−^ and aldehyde dehydrogenase 1 (ALDH1) in breast cancer cell line conferred stemness; which induced anoikis resistance through an increase in the phosphorylation of signal transducers and activators of transcription 3 (STAT3). The treatment with salinomycin, a selective agent, for targeting CSCs significantly reduced CD44^+^/CD24^−^, ALDH1 activity and STAT3 expression thus sensitized the cells to anoikis (62). Some studies reported the involvement of EMT in stemness of various cancers (129, 130), thereby facilitating aggressive tumor proliferation, anoikis resistance, and metastasis. Nevertheless, a genomic study reported three classes of CSCs: (i) glia CSCs with a high proliferation potential but repressed EMT markers; (ii) breast CSCs with high EMT signatures but less proliferative potential; and (iii) ovarian, prostate, and colon CSCs inhibited proliferative functions and varied EMT markers (131). Conversely, an experimental study reported the upregulation of MDA-9/syntenin in glioma stem cells fostered defensive autophagy and anoikis resistance (63). The blockade of MDA-9 promoted cell death through a reduced activation of the Bcl-2 and EGFR signaling (63). A recent study reported that low chaperone-mediated autophagy (CMA) enhanced the renewal of embryonic stem (ES) cell; however, CMA is upregulated at the onset of ES differentiation. The increase in CMA lowered intracellular α-ketoglutarate (α-KG) as CMA targeted isocitrate dehydrogenase 1/2 (enzymes decarboxylating isocitrate to yield α-KG) for lysosomal degradation, which in turn reduced the pluripotency and stemness of ES (64).

Despite the available literature highlighting the contribution of CSCs to tumorigenesis, a lot remains to be demystified. Future studies could consider understanding how various autophagy processes impact metabolism, thus mediating cancer stemness, anoikis resistance, and metastasis to improve the current therapy and overall survival of patients with cancer.

## Signaling Pathways as Drivers of Anoikis Resistance

The survival of all living organisms from unicellular to multicellular organism anchors on a cascade of signaling processes that involve the storage of energy and renewal of building blocks for cellular needs. Cancer cells are capable of altering metabolism based on the availability of nutrients and transcriptional factors, the level of free radicals or antioxidants, and activities of immune cells, thereby favoring energy needed for proliferation, anoikis resistance, and metastasis. We have discussed below some of the signaling pathways driving metabolic changes in cancer cells as targets for anoikis resistance among other factors (Figures 1, 2). An overview of the signaling pathways contributing to anoikis resistance is summarized in Table 1.

## Phosphoinositide-3-Kinase/Akt

The Phosphoinositide-3-kinase/Akt pathway is a crucial intracellular signaling pathway involved in cell proliferation and survival, migration, immune, and metabolic processes (132). Anomaly in this pathway is involved in the onset and progression of diverse diseases, including cancer, diabetes, neurological, and immunological disorders (133). PI3K is a family of enzyme that catalyzes the phosphorylation of phosphoinositide; its products can bind to other signaling kinases such as Akt and phosphoinositide-dependent protein kinase-1 to activate survival pathways (Figure 2).

Akt belongs to AMP/GMP kinases and protein kinase C-related family, and it can inhibit the activities of pro-apoptotic mediators, Bad, pro-caspase-9, and Fas-ligand. The target of rapamycin (TOR) is a central protein to life, and its mammal prototype called mTOR is an important regulator of protein synthesis and ribosome biogenesis. The upstream regulators of TOR include available nutrients, GFs, and their receptors to facilitate protein synthesis and other downstream processes necessary for life (134, 135). However, cancer cells exhibit an upsurge in this signaling pathway as it avails the signaling of oncogenic intermediates essential for continuous proliferation (135). Notably, phosphatase-tensin (PTEN), a negative regulator of the PI3K/Akt/mTOR pathway, acts as a tumor suppressor that inhibits growth and enhances cell sensitivity to anoikis (Figure 2) (65).

The elevated activity of PI3K/Akt is considered as a marker of cancer invasiveness (136). Similarly, activation of this pathway positively correlated with anoikis resistance in osteosarcoma (66, 67). A study reported activation of the PI3K/Akt pathway and the RAF/MEK/ERK pathway through caveolin 1 induced overexpression of IGF1R, thus promoting anchorage independence of hepatocellular carcinoma (HCC) (68). Also, PI3K/Akt activation through the downregulation of miR-133a-3p in prostate cancer was reported to promote anoikis resistance and bone metastasis (69). In addition, Akt signaling induced by stem cell-derived IL-8 promoted anchorage independence and pulmonary metastasis of osteosarcoma (70).

A cross talk exists between PI3K/Akt signaling and the MAPK/ERK signaling to promote cell survival, proliferation, and migration (137). The MAPK/ERK pathway, also known as the Ras-Raf-MEK-ERK pathway, plays a critical role in the initiation of cascades of events and conveys extracellular signals to intracellular targets (138). Anoikis-resistant ECs increased expression of RAS/ERK and PI3k/Akt, which facilitated ECM remodeling and invasiveness. Targeting either pathways impaired the proliferation, invasiveness, and sensitizated cells to anoikis (139). Similarly, upregulation of the MAPK/ERK pathway was reported in ECM-detached hepatoma although blockade of either ERK or PI3K/Akt had no significant effect in the sensitization of tumor cells to anoikis (71). On the contrary, a combined inhibition of both PI3K/Akt and ERK pathways sensitized the hepatoma to anoikis (71). The overexpression of Syndecan-2 in AIG melanoma cells was also reported to activate the PI3K/Akt and ERK signaling, hence promoting tumor invasiveness (41). This indicates some of the roles ECM components play in altering signaling pathways to promote anchorage independence and cancer cell invasive ability to circumvent anoikis. Mason et al. reported that oncogenic Ras enhanced the survival of ECM-detached cells with the aid of ATP generation in a PI3K and serum and glucocorticoid-regulated kinase-1 (SGK-1) dependent, but Akt independent, manner (26). The oncogenic Ras signaling promoted anoikis resistance by inhibiting the PH domain and leucine-rich repeat-containing protein phosphatase 1 (PHLPP1), which promotes anoikis through the activation of p38 MAPK. Despite the available literature on the role of PI3K in cancer, both at the basic research level and the clinical level (140), future studies on how anchorage-independence triggers its activation in various cancers are needed for better therapeutic strategies.

## Adenosine Monophosphate-Activated Protein Kinase

Adenosine monophosphate-activated protein kinase (AMPK) is a crucial cellular ATP and a nutrient sensor for regulating glucose, lipid, and cholesterol metabolic pathways to ensure the maintenance of energy homeostasis and nutrient availability as prompted by the level of AMP-ATP in the tissues (Figure 2) (141). An increased level of AMP allosterically activates AMPK, whereas an increased ATP level deactivates AMPK. AMPK can also be activated *via* phosphorylation by liver kinase B1 (LKB1) (Figure 2), calcium/calmodulin-dependent protein kinase 2, and TGF-β-activated kinase 1 (TAK1) (142).

Adenosine monophosphate-activated protein kinase is considered neither an ally nor an adversary in the progression of cancer (143), and the upregulation of PI3K/Akt pathway with concomitant inhibition of PTEN in most cancers leads to LKB1 impairment in the activation of AMPK for its tumor suppression role (144). Anoikis resistance was reported in LKB1-deficient lung cancer with an elevated glutamate dehydrogenase 1 (GDH1) that is associated with an increased α-KG, leading to CamKK2-mediated AMPK activation and the repression of mTOR activity (72). Other studies reported AMPK activation in cells following detachment from the ECM, thus facilitating anoikis resistance, and subsequent inhibition of AMPK restored the sensitivity to anoikis (73–75).

The detachment of cells from the ECM in breast cancer triggered activation of AMPK with concomitant repression of Akt phosphorylation, which enabled cell survival, leading to anoikis resistance and cell migration (76). However, once the cells were reattached, there was a decrease in the expression of AMPK that was mediated by Akt upregulation. This suggests a possible feedback loop between AMPK activity and Akt activity in the regulation of anoikis resistance and tumor metastatic potential capable of being explored in various cancers (Figure 2). On the contrary, AMPK activation using metformin in cholangiocarcinoma cell lines sensitized cells to anoikis *via* the inhibition of nuclear translocation, NF-κB and STAT3 (77). Hence, there is a need for additional studies to elucidate the role of AMPK in anoikis resistance and metastasis, especially for tumors with an aberrant AMPK basal expression, which might be a biomarker for diagnosis and guide in the choice of a targeted therapy.

## Hypoxia-Inducible Factors

Hypoxia-inducible factors are a family of proteins that are involved in response to oxygen unavailability (hypoxia) within the cell (145). Glioblastoma cultured in a poly hydroxyethyl methacrylate pre-coated dish presented with cell–cell detachment, spheroid formation, an upregulated HIF activity, a low ROS, and considerable resistance to anoikis (46). Silencing either HIF1α or HIF2α subjected HIF1α-targeted cells to anoikis but with no such effect recorded in HIF2α-targeted cells, thus elucidating the role of HIF1α in anoikis resistance (46).

The regulation of hypoxia-induced ANGPTL4 by HIF-1 enabled gastric cancer cell lines to achieve anoikis resistance and favor tumor growth (78). A study on HIF1 was reported to promote anoikis resistance through inhibition of integrinα5 in gastric cancer, and silencing of HIF1 sensitized the cells to anchorage dependence, impaired spheroid formation, increased the expression of integrinα5, and induced anoikis (79). HIF-1 was reported to be required by the oncogene ERBB2 for the dissemination of mammary gland tumors, anoikis resistance, and metastasis (80). The inhibition of HIF-1 in ERBB2 cells impaired ERK activity and Akt activity *via* the induction of pro-anoikis protein Bim (80). Taken together, the implication of the HIF family in promoting cancer development and anoikis resistance cannot be overemphasized as a result of the hypoxic environment. Nevertheless, studies and therapies targeted at manipulating the oxygen level in tumors may be an alternate path to potentiate death signals to overcome cancer proliferation and increase anoikis sensitivity.

## Signal Transducers and Activators of Transcription

A family of STAT includes STAT1, STAT2, STAT3, STAT4, STAT5a, STAT5b, and STAT6, which are located in the cytoplasm and are activated by cytokines (GFs, interferon, and interleukin), hormones, and ligands for their signal transduction in the nucleus (146, 147). In addition to their transcriptional function, anomalies of the STAT family had been established to increase susceptibility to various diseases and their progression (148). STAT5a was reported to play a significant role in the normal morphology of mouse prostate, and STAT5a null mice had epithelial cell deformation that elucidate the involvement of STAT5a in normal prostate formation (149).

Targeting STAT4 through siRNA intervention intensified anoikis in ovarian cancer, thereby impaired cell migration and invasion potential (81). The authors demonstrated that the overexpression of STAT4 heightened metastasis and reduced survival of tumor-bearing mice through the stimulation of cancer-associated fibroblasts (CAFs) from normal fibroblasts triggered by Wnt7a signaling (81).

STAT3 expression was reported to be upregulated in anoikis-resistant human pancreatic cancer cells (82). Silencing of STAT3 activity propelled anoikis; however, the overexpression of IL-6 and STAT3 enhanced anoikis resistance and protected the cells from piplartine treatment. Piplartine-pretreated cells failed to initiate tumor formation and metastasis, when injected subcutaneously or intravenously respectively, whereas non-treated anoikis-resistant cells had a high STAT3 expression and developed lung metastasis (82). The loss of Erbin, a ErbB2 (v-erb-b2 avian erythroblastic leukemia viral oncogene homolog 2) interacting protein activity in cervical cancer, was reported to upsurge STAT3 phosphorylation, thus facilitating anoikis resistance and tumor invasiveness *in vitro* and *in vivo* (83). Also, silencing MMP-2 in glioma cell lines impaired α5, β1 integrin, IL-6 signaling, and STAT3 phosphorylation, thus hindering the proliferation and growth of glioma (84). IL-6 stimulation of STAT3 was impaired as a result of salinomycin treatment on triple-negative breast cancer (TNBC) cells cultured in suspension, which inhibited stemness, MMP-9, and MMP-2 expression, leading to anoikis (62). The role of STAT family invariably in altering the ECM architecture is imperative to facilitating anoikis resistance and cancer progression (Figure 2). Future studies could elaborate on STAT family activities to proffer more effective strategies for cancer treatment.

## Rho GTPase

Rho GTPase belongs to the guanosine-5′-triphosphate (GTP) -binding protein family, a subfamily of the Ras superfamily that regulates diverse cellular activities, such as transcriptional processes, membrane trafficking, cell adhesion, cytoskeleton organization, and cell growth (150, 151). Guanine nucleotide exchange factors (GEF) and GTPase-activating proteins (GAP) are regulators of the Rho GTPase family (152). Rho GTPases are signaling proteins that regulate the catalysis and facilitate hydrolysis of GTP to GDP (guanosine diphosphate) and are active or inactive when bound to GTP or GDP respectively. Rho GTPases bind effector proteins [such as Rho-associated kinases 1 and 2, Rho-associated protein kinases (ROCKs) 1 and 2] and play a crucial role in reorganizing the actin cytoskeleton to promote cell migration (150, 153). Rac1, a Rho GTPase, was reported to enhance the transcription of estrogen receptor-alpha (ERα) in breast cancer. Targeting Rac1 pharmacologically inhibited estrogen-induced cell proliferation and attenuated the ERα transcriptional activity (85). Roc-A, a phytochemical, was reported to restrict cancer cell migration by inhibiting Rho GTPases, namely RhoA, Rac1, and Cdc42 activity, thereby changed cell morphology (86). Another study showed that the overexpression of ROCK 2 in colon carcinoma cells which were inoculated into immunocompromised mice altered the epithelial morphology and promoted tumor cell invasiveness into the surrounding tissues (154). Also, targeting ROCK 1 and ROCK 2 blocked medulloblastoma proliferation through the impairment of EMT, TGF-β, and TNFα *via* NFkβ signaling (87), which suggests that blockade of ROCK signaling could be a target to impair anoikis resistance and metastasis. Although the role of Rho GTPase in cancer was reviewed (150, 155), future studies are required to understand how targeting Rho GTPase can lead to the development of novel therapeutic agents for anoikis-resistant and metastasizing tumor cells.

## Targeting Metabolic Pathways to Ameliorate Tumor Metastases

The detachment of cells from the ECM is dependent on metabolic alterations (156), and metabolism describes all reactions involving the conversions and interrelationships of various biomolecules to achieve a balanced redox state, thereby supplying energy to support cell survival. The consequences of normal metabolism are healthy growth and development, while abnormal metabolism underlies diseases, including cancer. Reprogrammed metabolism is a characteristic of cancer cells for enhancing cell proliferation, migration, and invasion. Cancer cells can combine the normal pathway of glucose oxidation to yield pyruvate and employ aerobic glycolysis, whereby there is an increase in glucose uptake for lactate production, thus known as the Warburg effect (157). The glucose oxidation *via* either paths makes tremendous intermediates and ATP available for the synthesis of various biomolecules, which are used by cancer cells for their magnificent meshwork to minimize ROS production and to suppress host immunity, thus combating anoikis signals from detaching cells (158, 159). In addition to glucose, other biomolecules of significant importance for cell growth and anoikis resistance include proteins, lipids, and nucleotides (Figure 2). In this section, we discuss evidence from studies on the anoikis-resistance mechanism in relation to metabolic perturbation, an emerging regulatory path for targeting anoikis that remains to be fully explored (refer Table 1).

## Glucose Metabolism

The oxidation of glucose molecule to pyruvate occurring in the cytosol commences with the phosphorylation of glucose to glucose-6-phosphate (G6P) using hexokinase (Figure 2). This G6P is further converted into pyruvate in multi-catalyzed reaction steps to yield two molecules of ATP. This stepwise oxidation of glucose to pyruvate is termed glycolysis. Under extreme exercise conditions and in specific tissues (such as muscles, the brain, and the liver) with a limited oxygen supply, pyruvate can be rapidly converted to lactate by lactate dehydrogenase to meet the energy needs (160). On the other hand, oxidative phosphorylation (OXPHOS) generates about 36 ATPs per molecule of G6P. While the G6P-lactate pathway only generates about two molecules of ATP, it occurs spontaneously and efficiently, thus enabling both paths accumulate enough ATP needed for cancer cell growth and proliferation (161). The G6P-lactate reactions account for a good proportion of ATP used by cancer cells for their proliferative machinery, the Warburg effect. This enables cancer cells to circumvent production of ROS from the OXPHOS path, which is detrimental to the process of dysregulation of diverse signaling pathways culminating in acidosis of the tumor environment, uncontrolled cell growth, anoikis resistance, migration, and metastasis (42, 162). Highly invasive TNBC variant was demonstrated to exhibit metabolic perturbation as a result of utilizing glucose through the glycolytic path for its energy requirement in comparison to its less invasive variant; targeting these variants with 2-deoxy-glucose impaired glycolysis and sensitized more aggressive cell lines to anoikis (88).

Glycolysis-impaired cancer cells still survive, proliferate, and metastasize; cyclic adenosine protein kinase A (PKA) was identified to confer anoikis resistance in cancer cells in the presence of glucose starvation (89). The hyperactivation of PKA in anoikis-resistant cells stimulated autophagy and glutamine metabolism as alternate pathways for the energy needed through the abrogation of endoplasmic reticulum stress. The blockade of PKA activity impaired cancer cell survival and sensitized the cells to anoikis (89).

In addition to glycolysis, pentose phosphate pathway (PPP) is another pathway for glucose metabolism, called the hexose monophosphate shunt or the phosphogluconate pathway, which also takes place in the cytosol (Figure 2) with few similar intermediates to glycolysis, such as fructose-6-phosphate (F6P) and glyceraldehyde-3-phosphate (G3P) (163). PPP reaction is in two phases: first is the irreversible oxidative phase in which G6P is oxidatively decarboxylated to ribulose-5-phosphate (Ru5P) and nicotinamide adenine dinucleotide phosphate (NADPH); and the second is the reversible non-oxidative phase through which the Ru5P can be isomerized into ribose-5-phosphate (R5P), which either serves as a nucleotide precursor or is metabolized to yield F6P and G3P following the concerted enzymatic steps (164). Active division of cells require PPP to meet ribose-phosphate needs for DNA synthesis. Considering an intense metabolic requirement in cancer cells, PPP is a requisite for the proliferation of cancer cells to combat oxidative stress and to generate metabolites for various biosynthetic pathways (165).

Reduced nicotinamide adenine dinucleotide phosphate (NADPH), is an electron donor for cellular reactions such as nucleic acid, fatty acid, and steroids synthesis (166). The NADP^+^/NADPH ratio regulates glucose-6-phosphate dehydrogenase (G6PDH) activity in the cell. Cancer cells with a high proliferative activity have additional requirements for NADPH, glutathione (to mop free radicals from glutathione peroxidase activities), and G6PDH to supply DNA needs of pentose phosphates (164, 167), which highlights the importance of considering PPP intermediates as a potential target that could sensitize cancer cells to anoikis in future studies.

The growth of MDA-MB-231 cells in anchorage-independent conditions had increased ATP generation through fatty acid oxidation (FAO); it was observed that the glucose present in the cell was used to fuel PPP for redox homeostasis. The enhanced PPP activity subsequently fostered anoikis resistance in these cells (90). The treatment of MDA-MB-231 cells with a synthesized flavonoid (GL-V9) led to enhanced AMPK-activated lipid oxidation, ROS generation, limited PPP, and an increased sensitivity to anoikis (90). However, Mason et al. reported serum and glucocorticoid kinase-1 (SGK-1) role in energy availability for ECM detached cells, they elucidated it promoted increased glucose uptake via upregulated GLUT1 expression and PPP for increased ATP generation thus enabling anoikis resistance and survival (91).

Overall, the dysregulated glucose metabolism predisposes cancer cells to adopt resistance to anoikis and metastatic phenotypes (Figures 1, 2). Future studies could give considerable attention to elucidate glucose metabolic changes in various cancers for better therapeutic choices to improve the outcome of patients.

## Protein Metabolism

Similar to other biomolecules, protein is turned over in anabolism and catabolism to meet the carbon and nitrogen needs of the cell *via* its amino acid building blocks. These processes provide intermediates for several metabolic pathways to enhance cell growth, including cancer proliferation. The coiled-coil domain-containing protein 178 (CCDC178) is an 867 amino acid polypeptide that is mutated in several human cancers including gastric cancer (168) and HCC (169). CCDC178 was reported to be overexpressed in HCC and promoted metastasis by mediating anoikis resistance *via* activation of the ERK/MAPK signaling pathway (24). However, the depletion of CCDC178 inhibited ERK activation, which underlies the role of CCDC178 as a protein oncogene involved in anoikis resistance and cancer metastasis; therefore, identifying the potential of targeting this polypeptide in cancer treatment.

Recently, Kodama et al. reported an increased expression of c-Myc from the growth of cells in anchorage-independent conditions (92). The elevated expression of c-Myc correlated with the induction of phosphoribosyl pyrophosphate amidotransferase (PPAT), an enzyme responsible for the transfer of γ-nitrogen from glutamine to 5-phosphoribosyl pyrophosphate (PRPP), a key rate-limiting enzyme in the nucleotide biosynthetic pathway. A meta-analysis of most human cancers (considered by these authors) revealed a high ratio of PPAT/Glutaminase 1 (GLS1), which correlated with cancer prognosis. The depletion in PPAT suppressed growth in cancer cell lines cultured in 2D, 3D, and tumor progression *in vivo*, while its overexpression conferred malignancy (92). However, the overexpression of GLS1 impaired AIG, whereas its depletion, as reported in another study, did not affect tumor growth (170). These studies highlighted a shift in the glutamine metabolism that is capable of modulating AIG, anoikis resistance, and malignancy. Also, Weber discussed the changes in cancer metabolism at various stages of cancer progression (171). The author highlighted that an increase in glutamate metabolism in ECM-detached cells generated glycine and creatine necessary for *de novo* purine synthesis and ATP (171).

In glioblastoma, glutaminolysis was found to occur via the upregulation of glutamate dehydrogenase 1 (GDH1) (25). GDH1 catalyzes the conversion of glutamate into α-KG, thereby promoting the anaplerotic entry of glutamine intermediates to the TCA cycle (172). The knockdown of GDH1 impaired AIG by decreasing colony formation, cell proliferation, and tumor growth *in vivo* (25). Mechanistically, it was identified that EGFR phosphorylates ELK1 (a transcriptional activator in response to MEK/ERK pathway) to enhance GDH1 transcription and glutamine metabolism (25). Exploring the dynamics of glutamine metabolism in various cancers could be a promising approach to hamper the transformations fueling AIG, anoikis resistance, and metastasis.

Altogether, there is a need for future studies to elucidate how regulating protein metabolism impacts anoikis or its resistance.

## Fatty Acid Metabolism

Lipids are heterogeneous groups of biomolecules, which include fats, waxes, phospholipids, glycolipids, amino lipids, acyl lipids, and derived lipids. They are essential parts of diets, vitamins, hormones, cell membranes, and transport vesicles (173, 174). Lipids supply energy to the cells in a fasting state, supply essential nutrients for growth, provide thermal insulation outside the organs, coordinate different systems, provide a structural barrier support for cell membrane, and are involved in the transduction of signals (175, 176).

Acetyl-CoA is a central molecule for cellular metabolism, it is an intermediate from various biochemical processes (Figure 2), and acts as the building block for the biosynthesis of fatty acids and cholesterol and the end product of their oxidation (177). The synthesis of fatty acid is carried out in the cytosol to yield palmitate. Acetyl-CoA is carboxylated to malonyl-CoA in the presence of ATP, biotin, and bicarbonate, while acetyl CoA carboxylase, the rate-limiting enzyme, catalyzes this reaction (Figure 2). This step is followed by multi-catalyzed reactions *via* fatty acid synthase (FASN) complex with NADPH providing reducing equivalents for the production of palmitate which can be further esterified to yield complex lipids (178). In addition to the synthesis of *de novo* fatty acid, the requirement of cellular lipid can be achieved through exogenous fatty acid and amino acid uptake (179, 180).

Fatty acid oxidation, on the other hand, takes place in the mitochondria, and carnitine palmitoyltransferase 1 (CPT1) catalyzes the committed step of FAO. The cyclic cleavage of fatty acid from the carboxyl end of the chain is catalyzed by a fatty acid oxidase. Acetyl-CoA, the end product of FAO, enters the TCA cycle and the electron transport chain generate about 106 ATP and other metabolic intermediates per molecule of oxidized palmitate (177, 181).

Anomalies in lipid metabolism are associated with various diseases, including cancer (177, 182). Elevated FASN protein was found in the biopsy tissues of osteosarcoma patients presenting with lung metastasis in comparison with healthy individuals (183). It was reported that an increased expression of FASN mediated anoikis resistance and metastasis (94).

Some studies had highlighted FAO as a promoter of anoikis resistance and cancer progression (184, 185). Wang et al. reported the activation of FAO in colorectal cancer cells with an increased expression of CPT1, which correlated with anoikis resistance and metastasis *in vitro* and *in vivo* (95). Primary and metastatic tissues obtained from patients showed an elevated CPT1 in metastatic sites in comparison to the primary site corroborating the contribution of FAO to metastasis (95).

Similarly, breast cancer-expressing high Aldo-keto reductase family 1 member B10 (AKR1B10^high^) was reported to prefer FAO over glycolysis by utilizing exogenous fatty acids, thus favoring AIG and lung metastasis (96). However, the blockade of FAO in the AKR1B10^high^ cells impaired AIG and metastasis (96). Also, the utilization of FAO by TNBC enhanced metastatic relapse via depletion of cytoplasmic lipid droplets mediated by CUB-domain-containing protein 1 (97). Another study elucidated that accumulated lipid droplets enhanced anoikis resistance and invasiveness of cancer cells (43). Mechanistically, TGF-β2 signaling enabled fatty acid uptake for the storage of neutral lipids or catabolism of lipids to meet cellular ATP needs (Figure 2) (43).

Overall cancer cells utilization of lipid for cellular metabolism is mediated by factors such as; the presence of exogenous fatty acids (96), hormones signaling (186), adipocytes (187, 188), lipid droplets (97, 189), fatty acid receptors-transporters (190), energy status and intermediates from various metabolic paths (191). These factors determine the metabolic path activated or repressed based on cellular needs. Therefore, a holistic consideration of these factors is imperative for the exploration of lipid metabolism to enhance anoikis and improve the survival of patients with metastatic cancer.

## Nucleotide Metabolism

Nucleotides are biomolecules composed of nucleic acids as their building blocks, which are required for DNA and RNA synthesis. They are the major components of many coenzymes, and serve as the donors of phosphoryl groups found in some sugars or lipids. Nitrogen needs of the purine and pyrimidine synthesis are obtained from glutamine (Figure 2). Glutamine required for cellular growth can be either synthesized endogenously or obtained from the diet. Synthetic purine and pyrimidine analogs have been used as chemotherapeutic agents for cancer and other diseases (192). A recent study showed that high expression of glutamine synthase, an enzyme involved in the synthesis of glutamine, supports nucleotide metabolism, thereby leading to cancer cell survival (193). However, the role of nucleotide metabolism in mediating anoikis resistance is yet to be fully deciphered.

Rho-specific guanine nucleotide exchange factor (*Rgnef*) activated downstream of integrin in a complex with FAK, controls the migration of normal and tumor cells (194). In advanced-stage colon cancer patients, elevated expression, of *Rgnef* promoted tumor progression through its interaction with FAK (98, 99). Also, proteogenomic characterization of high-grade serous ovarian cancer cells revealed that the activation of RhoA pathway enhanced tumor metastasis and correlated with an impairment in the patient's survival (100). Recently, increased *Rgnef* expression was reported in the late-stage serous ovarian cancer, which was linked to tumor progression and a decrease in patient's survival (101). The deletion of *Rgnef* decreased the primary and metastatic tumor burden, elevated cellular ROS, inhibited AIG, thus reducing spheroid formation, and promoted anoikis (101). It is important to state that small molecules blocking oncogenic Rho signaling are in the early phase of development (195) and could be a promising approach for future studies aimed at regulating nucleotide-enhancing tumor metastasis.

## Perspective on Therapy to Target Anoikis Resistance

Since anoikis resistance is not required for survival by healthy cells, this occurrence is an attribute of metastatic cancer cells and, therefore, denotes a possible therapeutic target. However, the comprehensive mechanisms involved in anoikis resistance are not completely understood, and there are currently no anoikis resistance-targeting drugs available. We have discussed some mediators of anoikis resistance and how studies targeted the mediators to promote sensitivity to anoikis and hindered metastasis. Early diagnosis is important to identify and characterize specific anoikis-resistance markers, thereby targeting the same to improve anoikis and to prevent metastasis. Hypoxia and low pH are some of the major attributes of tumor milieu, enhancing anoikis evasion and therapy resistance. Several studies have shown that the therapeutic potential of small molecules (such as salinomycin) induced ROS production in cancer cells to inhibit anoikis resistance, invasiveness, migration, and metastasis (62, 90, 196–198). This suggested that unexplored drugs, which demonstrate their pharmacological targets by inducing oxidative stress capable of restricting AIG in cancer cells without affecting normal cells, may be considered for future therapies. In addition, an acidic environment is critical for the proliferation of solid tumors and has been reported to reprogram the metabolic landscape of the tumor microenvironment, thus promoting resistance to anoikis and metastasis (42). To circumvent the acidic microenvironment, the administration of bicarbonate for reducing cellular acidosis could be combined with other available therapies for the treatment of patients with metastatic cancer (111). Some pharmacological agents (such as metformin, piplartine, and 2-deoxy-d-glucose) that promote anoikis through the regulation of oncogenic or metabolic signaling pathways were reported, but the drugs are not specific for targeting anoikis-resistant cells.

## Discussion and Perspectives

Despite the available literature on the potential mediators of anoikis resistance preceding tumor metastasis, metabolic processes remain an emerging area of research that is currently less explored to enhance anoikis. UDP-glucose ceramide glucosyltransferase (UGCG), a key enzyme in glycosphingolipid metabolism, is overexpressed in metastatic breast cancer cells and augmented glutamine uptake for cellular energy metabolism (199). An increased expression of GLS enhanced oxidative stress response and glutamine oxidation to provide the required energy for cell proliferation (199). Metastatic breast cancer-overexpressing UGCG indicated that glutamine could be a precursor of other amino acid syntheses, such as aspartate and proline (199). An elevated proline biosynthesis contributed to tumor progression (200). Similarly, it was shown that Myc-dependent proline synthesis derived from glutamine mediated glucose oxidation *via* an increased extracellular acidification rate (ECAR) in human B lymphoma (P493) cells (200).

Another study reported that asparagine bioavailability correlated with the metastatic potential of patients with breast cancer (93). Genetic ablation of asparagine synthetase (ASNS), treatment with L-asparaginase, or restriction of dietary asparagine decreased metastasis without altering growth in the primary site whereas overexpression of ASNS or increased dietary asparagine reversed this effect. Similarly, the alteration in asparagine availability affected the invasiveness of cancer cells, which was associated with the regulation of EMT proteins (93). Thus, targeting asparagine metabolism could be a promising approach to prevent metastatic cancer cells and promote anoikis. Branched-chain amino acids (BCAAs), especially valine, leucine, and isoleucine, are essential amino acids obtained from the diet. Cancer cells have an enhanced ability for BCAA uptake (201). Also, BCAA aminotransferases (BCATs), the enzymes catalyzing the first step in the catabolic pathway of BCAAs, are overexpressed in cancer cells (202, 203). It was demonstrated that highly malignant pancreatic ductal adenocarcinoma (PDAC) cells relied on BCAA for its survival and proliferation, as well as a carbon source, to enhance lipogenesis in the cells (204). Genetic blockade of BCAT2 suppresses PDAC cell proliferation *in vitro* and *in vivo*. Considering these findings, exploring the regulation of BCAA catabolic pathways may enhance anoikis and improve the therapy for metastatic cancer cells.

Dietary methionine, an essential amino acid in one-carbon metabolism, has been reported to alter tissue metabolism (205, 206), and its depletion or restriction has been proposed to have an anticancer effect (207, 208). More recently, dietary methionine restriction altered methionine and sulfur metabolism in murine colorectal cancer *via* enhanced sensitivity to chemo- and radiotherapy (209). The restriction of methionine from the diet of healthy individuals had a profound impact on methionine levels and systemic metabolism. The methionine restriction affected metabolites of methylation, TCA cycle, amino acid, and nucleotide metabolism (210). These suggest regulating methionine dietary intake or systemic level in anoikis resistant or metastasizing tumor cells could be a promising approach to promote anoikis.

The upregulation of guanosine nucleotide was reported in a subtype of small-cell lung cancer (SCLC) (210). The tumor from this SCLC subtype expressed an increased guanosine biosynthetic enzyme known as inosine monophosphate dehydrogenase 1 and 2 (IMPDH1 and IMPDH2). Also, this subtype overexpressed Myc, which is a transcriptional target of IMPDH (211, 212), and targeting IMPDH drastically reduced tumor growth (210). In addition, Valvezan et al. identified that IMPDH inhibitor selectively targeted the cell and tumor models of tuberous sclerosis cells (TSCs) in an mTORC1-dependent manner (213). It was observed that mTORC1 promoted anabolic cell growth and proliferation *via* the regulation of ribosome biogenesis and nucleotide synthesis to sustain the anabolic balance, which indicated an increased dependence on the nucleotide synthesis in cells having an activated mTORC1. We suggest that the IMPDH inhibitors could be repurposed for the treatment of anoikis-resistant or metastatic cancer cells-overexpressing Myc. Phosphoribosyl-pyrophosphate synthetase 2 (PRPS2), a rate-limiting enzyme, in the purine synthesis was identified to promote the elevation of nucleotide biosynthesis in Myc-transformed cells (214). PRPS2 connected the nucleotide and protein biosynthesis through a specific cis-regulatory element within the PRPS2 5′ UTR. The strategies to block the PRPS2 expression or interfere with an Myc-dependent translational control hindered the availability of PPP and nucleotide intermediates, which led to a reduced nucleotide production and consequently slowed tumor initiation and proliferation without affecting the normal cell survival (214). The Myc oncogene remains undruggable but PRPS2 as an enzyme is considered a constituent of a druggable genome (215). Therefore, the drugs specifically targeting PRPS2 or other enzymes involved in nucleotide biosynthesis may be important for metabolic reprogramming in anoikis-evading cancer cells.

Gene expression is closely regulated at various levels, which includes transcription, posttranscription, translation, and posttranslation. Recently, a key nucleotide component, N^6^-Methyladenosine (m^6^A), emerged as a new layer of regulatory mechanism, which modulates gene expression in living organisms (216). Some studies showed the existence of m^6^A on several types of RNA [such as mRNA, rRNA, miRNA, lncRNA, circular RNA (circRNA), and small nuclear RNA (snRNA)], which was constantly regulated during normal development and disease states (inclusive of cancer) (216–218). The dysregulation in the m^6^A modification was reported in cancer pathogenesis and its response or resistance to drug treatment (219–221). Considering the critical role of RNA modification in cancer, there is a need for better understanding of the underlying mechanisms of these modifications and exploring such potential therapeutic targets to circumvent anoikis resistance.

## Concluding Remarks

A few studies had shown several factors that confer the survival of cancer cells after detachment of cells from the ECM. These factors enable cancer cells to evade anoikis, thus supporting the formation of metastasis in secondary sites. Recent evidence revealed that aberrations in the ECM (5) are mediated by specific upregulated metabolic genes or intermediates, and transcriptional signaling pathways that drive acidic environment, EMT, and oncogenic processes (Figures 1, 2). Importantly, metabolism reprogramming, a hallmark of cancer, is critical to ECM detachment; however, our knowledge from literature is limited on the impact of manipulating a single metabolic pathway or the overall metabolic pathways in metastasis. A holistic approach of maintaining ECM stability to facilitate pro-apoptotic pathways for the eradication of detached cells may be relevant for enabling anoikis and achieving a successful therapy. Hence, a better understanding of the metabolic changes enhancing anoikis resistance could unravel novel approaches to target metastasis, the leading cause of cancer-related deaths.

## Author Contributions

FA and GZ conceived the idea. FA wrote and revised the manuscript. GZ and XW reviewed and supervised the writing process. AA contributed significantly to the writing and figures design. LA and DY gave helpful suggestions throughout the writing. All authors contributed to the article and approved the submitted version.

## Conflict of Interest

XW is a member of the advisory board of the BinDeBioTech Co., Ltd. The remaining authors declare that the research was conducted in the absence of any commercial or financial relationships that could be construed as a potential conflict of interest.
